# First Proof-of-Principle of PolyJet 3D Printing on Textile Fabrics

**DOI:** 10.3390/polym15173536

**Published:** 2023-08-25

**Authors:** Tomasz Kozior, Andrea Ehrmann

**Affiliations:** 1 Faculty of Mechatronics and Mechanical Engineering, Kielce University of Technology, 25-314 Kielce, Poland; tkozior@tu.kielce.pl; 2 Faculty of Engineering and Mathematics, Bielefeld University of Applied Sciences and Arts, 33619 Bielefeld, Germany

**Keywords:** 3D printing, adhesion, PolyJet modeling (PJM), composite, woven fabrics

## Abstract

Possibilities of direct 3D printing on textile fabrics have been investigated with increasing intensity during the last decade, leading to composites which can combine the positive properties of both parts, i.e., the fast production and lateral strength of textile fabrics with the flexural strength and point-wise definable properties of 3D printed parts. These experiments, however, were mostly performed using fused deposition modeling (FDM), which is an inexpensive and broadly available technique, but which suffers from the high viscosity of the molten polymers, often impeding a form-locking connection between polymer and textile fibers. One study reported stereolithography (SLA) to be usable for direct printing on textile fabrics, but this technique suffers from the problem that the textile material is completely soaked in resin during 3D printing. Combining the advantages of FDM (material application only at defined positions) and SLA (low-viscous resin which can easily flow into a textile fabric) is possible with PolyJet modeling (PJM) printing. Here, we report the first proof-of-principle of PolyJet printing on textile fabrics. We show that PJM printing with a common resin on different textile fabrics leads to adhesion forces according to DIN 53530 in the range of 30–35 N, which is comparable with the best adhesion forces yet reported for fused deposition modeling (FDM) printing with rigid polymers on textile fabrics.

## 1. Introduction

Additive manufacturing, or 3D printing, enables forming new shapes which are often not accessible by common technologies, and working without molds, which favors individualization [1,2,3]. Amongst the diverse 3D printing techniques, fused deposition modeling (FDM) is most often used, while stereolithography (SLA), selective laser sintering (SLS), or selective laser melting (SLM) are also well known and used for specific materials and applications from the medical and biotechnological field, dealing with polymeric stents and substrates for tissue engineering to metallic parts of microsatellites [4,5,6].

In spite of the aforementioned advantages of 3D printing, there are also disadvantages which should be mentioned, such as long printing durations, an often undesired anisotropy due to the layer-by-layer building, and generally reduced mechanical properties compared to die casting [7,8,9]. One possibility to overcome these problems is provided by combining 3D printing with textile substrates, forming, in this way, a composite with tailorable mechanical properties in-plane and out-of-plane [10]. Corresponding studies are mostly reported for FDM printing on different textile fabrics, discussing different applications such as filters, functional textiles, or fashion [11,12,13]. On the other hand, the adhesion between both parts necessitates optimization of diverse material and printing parameters [14,15,16], especially since the molten FDM polymer has a relatively high viscosity and thus does not easily build a form-locking connection with the textile substrate.

Resins with much lower viscosities are used, e.g., in SLA printing. A study working on SLA printing on textile fabrics has proven the principle feasibility of SLA printing for the formation of a textile-resin composite, but has also mentioned the problematic mounting of the textile below the printing bed and the necessity of washing the unpolymerized resin fully out of the textile, making this technique not ideally suited to produce polymer/textile composites, either [17].

An optimum technique to produce 3D print polymers on textile fabrics would apparently combine printing from the top, with the material being placed only at the desired positions with a low-viscous printing material, i.e., a resin or an ink. This combination can be found in PolyJet modeling (PJM) where liquid photopolymers are jetted onto defined positions of a substrate and UV-hardened there [18]. The great advantage of using PJM technology to build composite models in combination with textiles is the possibility of using several different materials (rigid and soft) as well as mixing them to build a new one. Among the available PJM materials, there are the medical materials MED610 and 620, which are biocompatible for medical and dental purposes [18]; Agilus30, with very high tear resistance; VeroDent materials, especially for dental materials; and Digital ABS plus for tools and electrical parts production, among many others [19]. In addition, 3D PJM printing allows models to be produced from different materials during the construction of one model, which allows an object to be created with variable properties in its different places. This may allow for proper dosing of a specific material in those places where the model will be combined with textile materials. Another advantage of this technology is the possibility of printing models from transparent materials, which increases the practical use in industrial applications.

Here, we report the first proof-of-principle of PJM printing on textile fabrics along with its advantages and the problems, which have yet to be solved.

## 2. Materials and Methods

The textile fabrics used for these tests are a polypropylene (PP) nonwoven (29 g/m^2^) fabric, a linen woven fabric (196 g/m^2^), a cotton woven fabric (143 g/m^2^), two polyester woven fabrics (116 g/m^2^ and 127 g/m^2^, the latter roughened on one side), and different warp-knitted polyester fabrics (9 g/m^2^, 65 g/m^2^, and 248 g/m^2^, respectively). Microscopic images of all fabrics under investigation are shown in Section 3.

Printing was performed using the PJM printer Connex 350 (Stratasys, Eden Prairie, MN, USA), using the resin Fullcure720 (Stratasys,) and the support material FullCure705 liquid resin (Stratasys) for the first layers. The polymerized Fullcure720 resin has the following properties according to the data sheet: tensile strength of 60.3 MPa, elongation at break of 15–25%, modulus of elasticity of 2870 MPa, flexural strength of 75.8 MPa, flexural modulus of 1718 MPa, compression strength of 84.3 MPa, shore hardness of 83 D, and glass transition temperature of 48.7 °C.

Sample dimensions are 50 mm × 50 mm × 1.5 mm of Fullcure720, partly with an additional 0.5 mm of support material below. During the printing process, the high speed mode was used with a set layer thickness of 0.032 mm.

**Figure 1:**
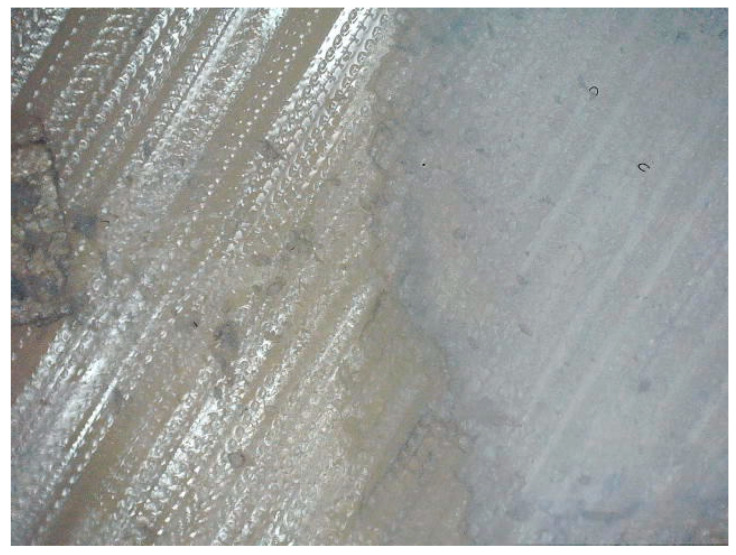
Support material Fullcure705 (on the left side of the image, yellowish material with visible drops of material) partly scratched away from the main printing polymer Fullcure720 (on the right side of the image, white material). The long image size corresponds to 1.25 mm.

It must be mentioned that printing with PJM technology means that, firstly, several layers of a support material are printed before the main building material is used to print the actual model. The support material, however, is not mechanically stable, as can be seen in Figure 1. Here, a layer of support material, Fullcure705, on top of Fullcure720 was simply scratched away with a fingernail, making the main polymer visible (on the right side of either image).

Since it is thus not the best choice for the proof-of-principle, besides normal printing with the support material as first layers in contact with the textile fabric, a second test series was printed with the main material Fullcure720 in contact with the textile fabric.

Modification of the G-code is not possible for the used printer. Thus, the process was stopped after printing the raft support layers (0.5 mm) on the common substrate, the raft was removed, the textile fabric attached on the building platform, and printing with the main material was started.

Adhesion tests on samples with 25 mm width were performed using a Sauter FH2K universal test machine according to DIN 53530 [20] and evaluated according to ISO 6133 [21], procedure B, taking into account the median of the measured adhesion force peaks for each sample. Microscopic images were taken by a Camcolms2 (Velleman, Gavere, Belgium) digital microscope.

## 3. Results and Discussion

**Figure 2:**
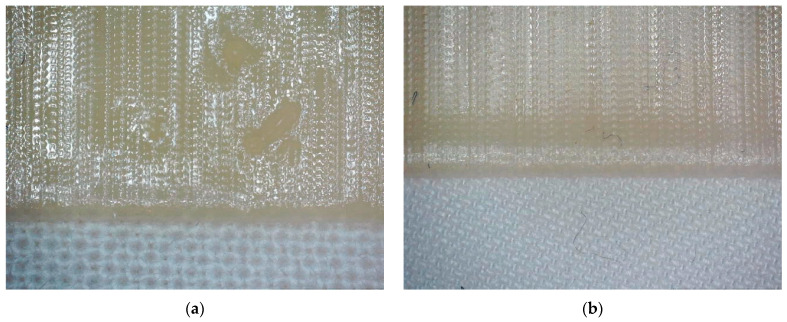

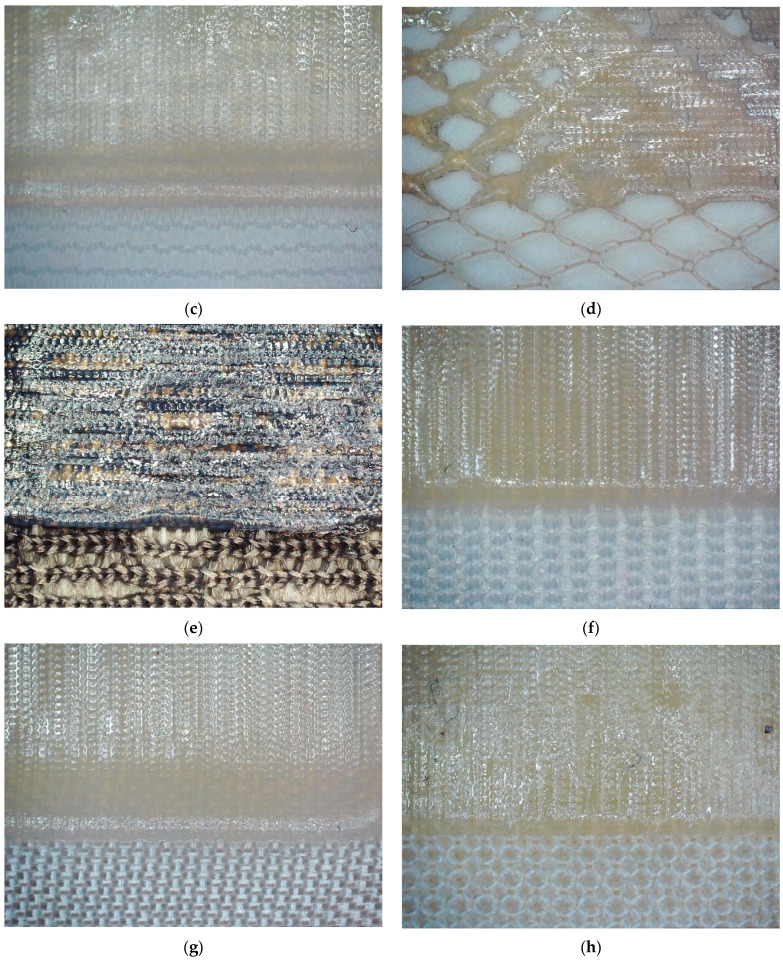
Fullcure705 printed on different textile fabrics: (**a**) cotton; (**b**) roughened polyester; (**c**–**f**) warp-knitted fabrics 1–4; (**g**) polyester; (**h**) linen. The long image sizes correspond to 1.25 mm.

Due to the similar viscosities of both resins, the first tests were performed with the support material Fullcure705 to investigate the principle possibility to perform PJM printing on different textile samples. The results are depicted in Figure 2.

Generally, PJM printing with Fullcure705 was possible on nearly all samples under investigation. In most images, a closed polymer layer is visible on top of the fabric. An exception is visible in Figure 2d, where the pores of the warp-knitted fabric #2 were partly too large to serve as a substrate for the low-viscous printing polymer. Obviously, PJM printing can only work on relatively closed fabrics, opposite to the highly-viscous FDM strands which are also able to span larger pores in textile fabrics. In Figure 2, in particular in Figure 2a, defects related to the 3D printing process on a textile material are visible. This is due to the fact that this technology currently does not easily enable 3D printing on existing objects, and the process presented in this article is the first experimental study reported in this area.

The thin (0.5 mm) raft from this support material clearly shows the printing direction, sometimes with small holes, which may be due to morphological modifications in the textile fabrics below. Opposite to FDM printing on textile fabrics, PJM printed resin cannot be expected to level out substrate height variations. This is slightly visible for the warp-knitted fabric #3 (Figure 2e), which has the strongest morphological variation amongst the chosen fabrics.

**Figure 3:**
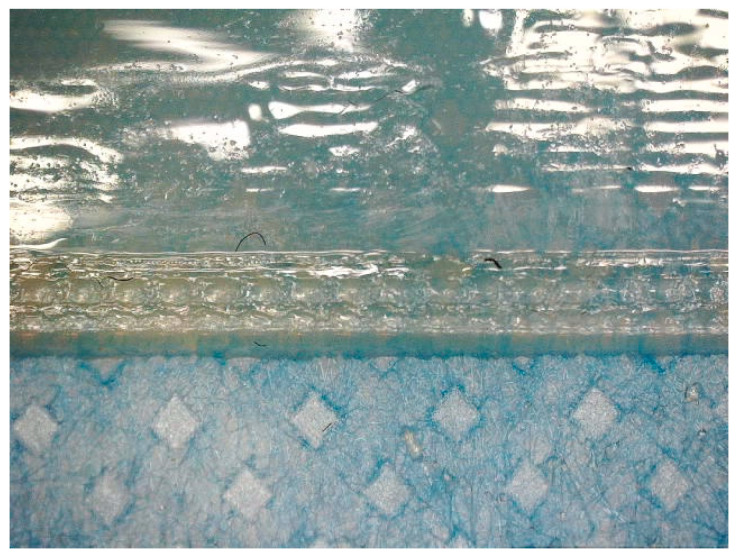
Fullcure720 (shiny area at the top) printed on a raft from Fullcure705 (visible in the middle as the border of the raft), printed on a PP nonwoven (blue part at the bottom). The long image size corresponds to 1.25 mm.

Mechanical investigations cannot be performed with this resin due to its brittleness. As the next test, Fullcure720 was thus printed on the support material Fullcure705, which formed the raft printed on a PP nonwoven, as depicted in Figure 3.

However, trying to detach the Fullcure720 layer from the textile resulted in detaching it from the Fullcure705 layer, which stayed on the textile fabric. In this way, it is not possible to gain any adhesion between textile fabric and the main polymer layer, since both are not directly in contact, but separated by the raft.

**Figure 4:**
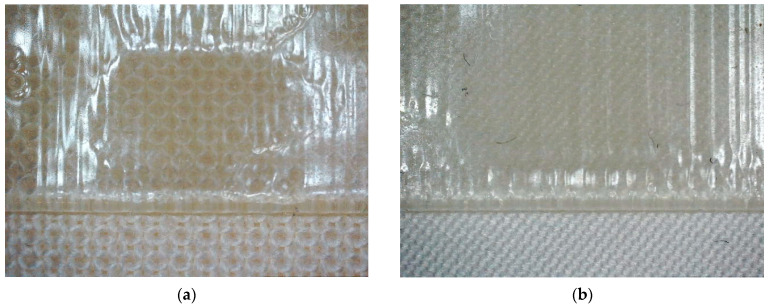

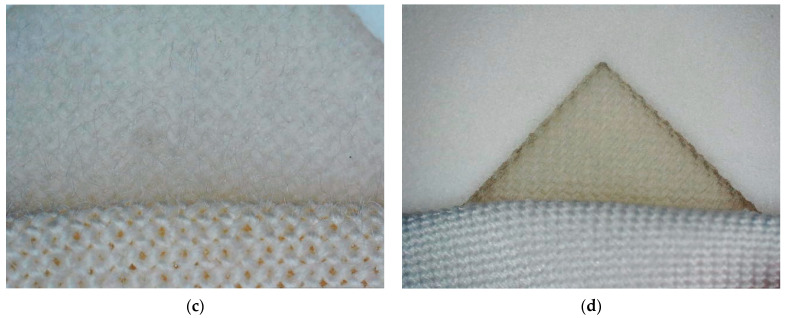
Fullcure720 printed on (**a**) linen, and (**b**) roughened polyester. Back of the printed material after partly detaching it manually from the textile fabrics below, showing (**c**) linen (polymer with fibers in the upper part, detached fabric in the lower), and (**d**) roughened polyester (triangular corner of the polymer after detaching the fabric visible in the lower area). The long image sizes correspond to 1.25 mm.

As described in Section 2, in the next test series, the raft was thus printed on the printing bed, the printer was stopped, the raft was removed, the textile mounted on the printing bed, and the main polymer was printed directly on the textile fabric. Some of the results are depicted in Figure 4.

As expected, the surface of the main materials looks much smoother than that of the support material (also visible in Figure 3), and the textile below is well visible through the 1.5 mm thick polymer layer (Figure 4a,b). Manually detaching the polymer layer from the textile fabric also necessitated some force, and the back of the polymer layers showed residues of textile fibers (Figure 4c,d), similar to well-adhered FDM prints on textile fabrics.

**Figure 5:**
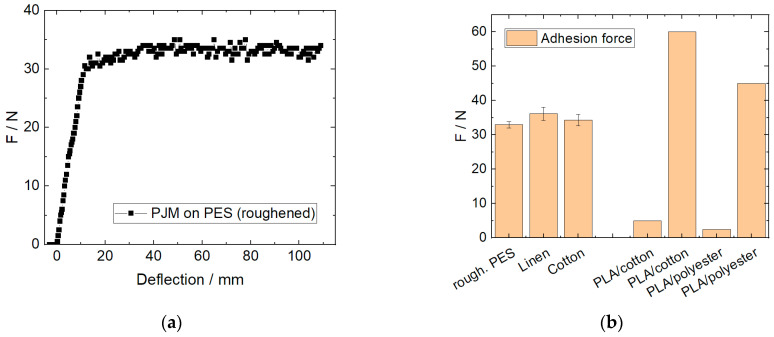
(**a**) Adhesion measurement of Fullcure720 printed on a polyester fabric; (**b**) adhesion forces for the tested material combinations. A few exemplary values from fused deposition modeling (FDM) printed poly(lactic acid) (PLA) are added (maxima and minima values for the respective material combinations taken from [22,23]).

Next, adhesion tests were performed on the samples printed with Fullcure720 on cotton, linen, and roughened polyester. Figure 5a depicts an exemplary adhesion measurement according to DIN 53530, while Figure 5b shows evaluations of the adhesion forces for PJM printing on the aforementioned materials in comparison with the literature values [19,22] regarding identical adhesion tests of poly(lactic acid) (PLA) strips, printed with fused deposition modeling (FDM) on cotton and polyester, respectively. While some literature results show higher adhesion forces than those gained here, there are many reports of much smaller adhesion forces. This comparison shows that without any optimization, PJM printing on different textile fabrics reaches comparable adhesion forces as those reached by optimized FDM printing processes with PLA, which is known to have the highest adhesion forces among the common rigid FDM printing polymers [22].

**Figure 6:**
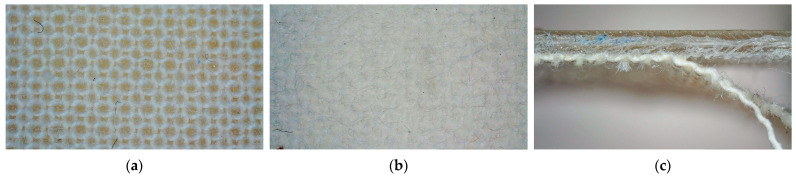
(**a**) Back of the linen fabric with penetrating resin visible in the pores; (**b**) detached linen fibers on the back of the print after adhesion test; (**c**) linen fabric partly detached from the imprinted resin. The horizontal image size is 10 mm.

Microscopic photographs after the adhesion tests are depicted in Figure 6. Before the adhesion tests, the resin is visible inside the pores on the back of the linen fabric (Figure 6a). After adhesion, linen fibers are stuck on the back of the print, underlining the good adhesion between both components (Figure 6b). Figure 6c depicts the border between fixed and detached fabric below the resin.

Nevertheless, it must be mentioned that the low degree of freedom, regarding modifications of the G-code, make PJM printing unnecessarily complicated and inefficient. Moreover, if the pores in the fabric are too large, the resin will strongly stick on the building platform (which is no longer protected by the detachable raft). Finally, it is necessary to carefully fix the textile fabric on the printing bed to avoid collisions with the roller, which aligns each applied polymer layer before photopolymerization. It seems that the problem of printing on already existing models and materials can be solved by, for example, using special holders, as is the case with conventional machining.

When these technical problems are solved, more and larger samples can be printed to perform quantitative adhesion tests as well as tests regarding their stab resistance, since printing a scale-like structure on textile fabrics belongs to the possibilities to produce lightweight, flexible body armor.

## 4. Conclusions

PolyJet modeling (PJM) was reported for the first time as suitable for direct 3D printing on textile fabrics. Only samples with large pores could not be imprinted with this technique due to the low viscosity of the used resins flowing through the fabric. Both the support material Fullcure705 and the main printing polymer Fullcure720 could successfully be printed on diverse textile fabrics. The next experiments will include quantitative adhesion tests, depending on textile fabrics, printing materials, and printer settings. MED610 as a biocompatible resin will also be investigated to enable future medical or dental applications of the new polymer/textile composites.

3D printing with the use of PJM technology on textile objects is a complicated process and requires an appropriate manufacturing strategy and selection of technological parameters, as there are no guidelines from the manufacturer of 3D printers in this area, which is currently the subject of further research by the authors of the article.

## Data Availability

All data are included in this paper.
